# The burden of alcohol, tobacco and others drugs among incarcerated population diagnosed with tuberculosis: time trends and spatial determinants in Southern Brazil

**DOI:** 10.1186/s12889-022-13408-1

**Published:** 2022-05-17

**Authors:** Alessandro Rolim Scholze, Josilene Dália Alves, Thaís Zamboni Berra, Felipe Lima dos Santos, Antônio Carlos Vieira Ramos, Giselle Lima de Freitas, Maria José Quina Galdino, Flávia Meneguetti Pieri, Marcos Augusto Moraes Arcoverde, Sandra Cristina Pillon, Aline Aparecida Monroe, Inês Fronteira, Dulce Gomes, Ricardo Alexandre Arcêncio

**Affiliations:** 1 Department of Maternal-Infant and Public Health Nursing, Ribeirão Preto College of Nursing, University of São Paulo, São Paulo, Ribeirão Preto Brazil; 2 Institute of Biological Sciences and Health, Federal University of Mato Grosso, Barra do Garças, Mato Grosso Brazil; 3 Department of Maternal-Infant and Public Health Nursing, College of Nursing, Federal University of Minas Gerais, Belo Horizonte, Minas Gerais Brazil; 4 Department of Nursing, State University of Northern Paraná, Bandeirantes, Paraná Brazil; 5 Department of Nursing, State University of Londrina, Londrina, Paraná Brazil; 6 Department of Nursing, West Paraná State University, Foz do Iguaçu, Paraná Brazil; 7 Department of Psychiatric Nursing and Human Sciences, Ribeirão Preto College of Nursing, University of São Paulo, São Paulo, Ribeirão Preto Brazil; 8 Global Health and Tropical Medicine, Instituto de Higiene E Medicina Tropical, Universidade Nova de Lisboa, Lisbon, Portugal; 9 School of Science and Technology, Research Center in Mathematics and Application, University of Évora, Évora, Portugal

**Keywords:** Tuberculosis, Prisoners, Drug utilization, Public health

## Abstract

**Background:**

Tuberculosis (TB) is an infectious disease caused by *Mycobacterium tuberculosis* and is a public health problem worldwide. It is estimated that 90% of the patients diagnosed with TB live in vulnerable environments with limited health resources, such as individuals living in correctional facilities. This study aimed to identify the consumption of alcohol, tobacco, and other drugs among prisoners diagnosed with TB and the spatial determinants and time trends of the phenomenon in southern Brazil.

**Methods:**

A cross-sectional study using data from the Brazilian Notifiable Diseases Information System was carried out. TB cases confirmed from 2014 to 2018 in prisons located in Paraná, Brazil, were selected. The Prais-Winsten procedure was performed to identify time trends by calculating monthly rates and the percentage of monthly variation. The Seasonal-Trend by Loess decomposition method was used to verify the time series and trends. The spatial association was verified with the Getis-Ord Gi* technique, and the risk areas were identified using spatial scan statistics.

**Results:**

A total of 1,099 TB cases were found in the studied population. The consumption of tobacco (*n* = 460; 41.9%), illegal drugs (*n* = 451; 41.0%), and alcohol (*n* = 179; 16.3%) stood out. An ascending trend was found for the consumption of alcohol (+ 19.4%/mo. (95%CI: 12.20–23.03)), tobacco (+ 20.2%/mo. (95%CI: 12.20–28.82)), and illegal drugs (+ 62.2%/mo. (95%CI: 44.54–81.97)). Spatial analysis revealed clusters for the use of alcohol, tobacco, and illegal drugs.

**Conclusions:**

This study advances knowledge presenting the burden of drug use and its typology among individuals diagnosed with TB in the prison system. There is a growing trend among patients to use drugs, especially illegal drugs. The clusters show differences between the places where the prisons are located.

## Background

Tuberculosis (TB) is an infectious disease caused by *Mycobacterium tuberculosis* and is a public health problem worldwide, although developing countries are the most severely affected [1]. Data show that 30 countries account for 87% of the disease burden, and Brazil ranks 19^th^ among them [2].

Even though the TB burden declined in recent years before the COVID-19 pandemic [3], it is a disease difficult to control and eliminate due to co-infection with human immunodeficiency virus (HIV); antimicrobial resistance; multidrug-resistant TB; and increased consumption of alcohol, tobacco, and/or other drugs. In addition, TB is associated with social determinants of health, such as social vulnerability, poverty, and social exclusion to which populations such as immigrants, refugees, the homeless, and the incarcerated population are exposed [4]. It is estimated that 90% of the patients diagnosed with TB live in vulnerable environments with limited health resources [5], such as individuals living in correctional facilities.

The World Health Organization (WHO) estimates that the prevalence of TB among incarcerated population is 100 times higher than in the general population [6–8]. The prison environment contributes to TB mortality rates, revealing that the control of the disease is a priority neglected throughout the world [6, 7, 9]. It is possible to note that studies that address TB in the incarcerated population are still incipient, as it was not possible to show descriptive statistics that describe TB mortality within prison environments. What can be pointed out is that prisons act as a reservoir for infection and transmission of the bacillus, and prisoners are a high-risk population for TB infection [10].

The insalubrity to which this population is exposed should also be considered. Brazilian prisons are considered an important reservoir of the TB bacillus, and consequently, a source of transmission between prisoners, correctional officers and their families [11].

A literature review conducted with the following descriptors “incarcerated”, “drug abuse and tuberculosis”, and “temporal trends and clustering”, revealed that few studies address the use of alcohol, tobacco, and other drugs among the incarcerated population diagnosed with TB, showing a need for further research. The development of studies addressing this population is crucial to promote equity and mitigate the effects of the TB burden, especially in a country like Brazil with a large contingent of prisoners. Therefore, this study’s objective is to identify the consumption of alcohol, tobacco, and other drugs among prisoners diagnosed with TB and the spatial determinants and time trends of the phenomenon in southern Brazil.

## Methods

### Study design

Cross-sectional study [12].

### Study setting

**Fig. 1:**
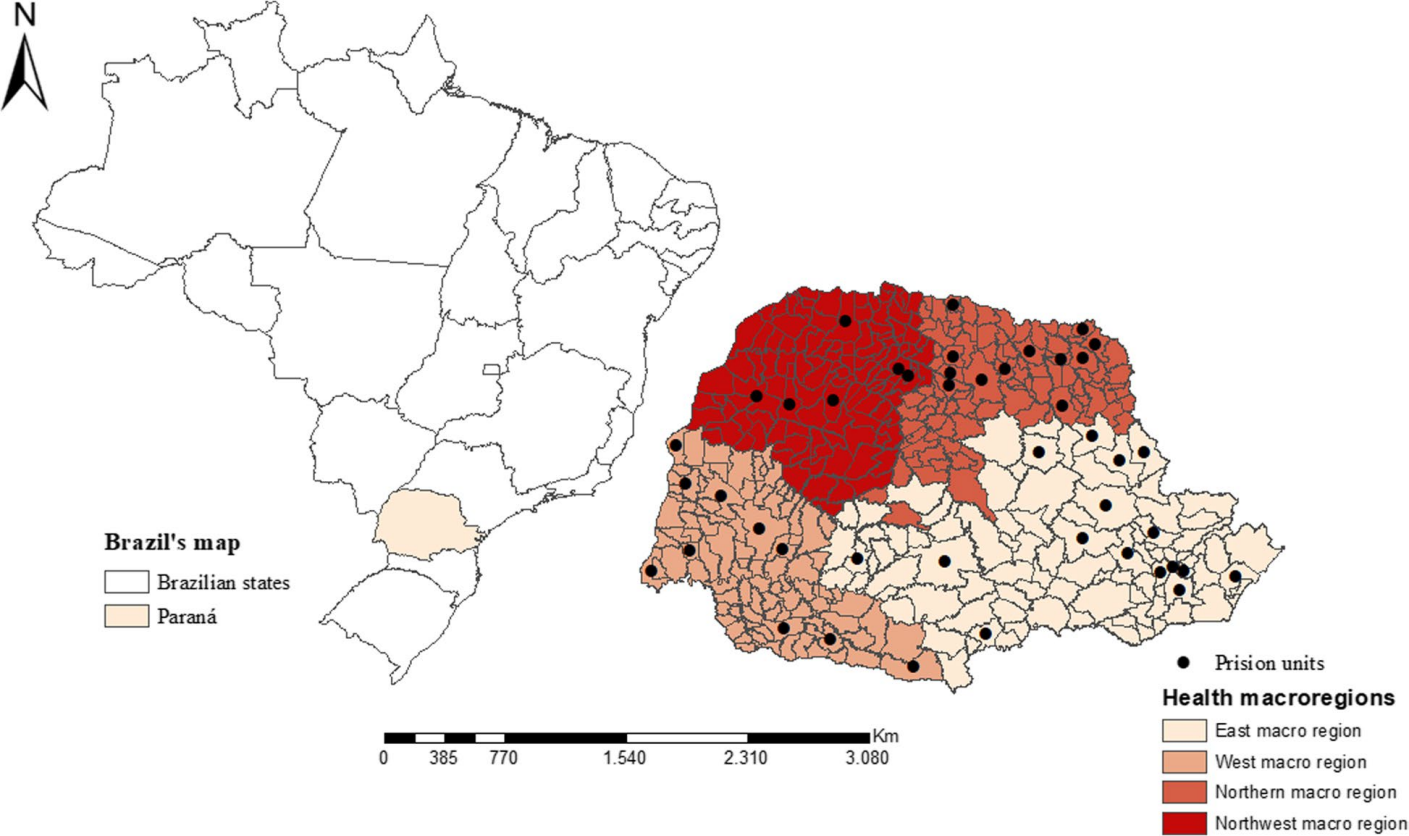
Distribution of macro-regions and prisons in the cities located in Paraná, Brazil (2021)

This study was conducted in the state of Paraná (in southern Brazil), which is divided into four health macro regions (east, west, north, and northwest) with 399 cities and an estimated population of 11.34 million inhabitants. It is noteworthy that, in the state of Paraná (399 municipalities in all), there are 63 prison units distributed in 44 municipalities in the state, and all were included in the study [13]. Figure 1 shows the spatial distribution of prisons in the state of Paraná according to the four health macro regions.

### Population and study period

It is estimated that Brazil has an incarcerated population of 748,009 individuals [13], ranking third among the countries with the largest number of prisoners, with an incidence of 352.6 prisoners per 100,000 inhabitants [14]. Paraná has the seventh largest proportion of incarcerated population in the country, with a population of approximately 29,831 [13].

The TB cases confirmed in the state’s prisons from 2014 to 2018 and reported to the Notifiable Diseases Information System (SINAN), Brazilian Ministry of Health, were selected. The SINAN collects, reports, and disseminates data concerning reportable diseases according to information provided by the epidemiological surveillance agencies of cities.

Data were collected from the state Health Department using an electronic spreadsheet. The data are anonymous, and patients cannot identified by name or facility. Inclusion criteria were the incarcerated population diagnosed with TB from 2014 and 2018 using alcohol, tobacco, or illegal drugs. The period between 2014 and 2018 was chosen because the reporting form was updated in 2014 and started including information regarding the incarcerated population and drug use.

It is noteworthy that illicit drugs are those whose sale is prohibited and enter the country illegally, such as marijuana, cocaine, crack, ecstasy, LSD, inhalants, heroin, barbiturates, morphine, skank, mushroom tea, amphetamines, chloroform, opium and others [15].

### Data analysis

The database was checked for consistency, and the cases were grouped according to the following characteristics: 1) total incarcerated population considering all TB confirmed cases; 2) incarcerated population consuming alcohol or reporting to be alcoholic; 3) incarcerated population using tobacco or reporting to be a smoker, and 4) incarcerated population using illegal drugs. Note that the same patient may use more than one drug and be included in more than one group.

### Analysis procedures

#### Exploratory

Descriptive statistics were performed to present the absolute and relative frequencies of the variables sex, age, race, education, TB/HIV co-infection, diabetes mellitus, mental disorders, and information regarding TB clinical profile (such as admission, type, and whether the following were performed: radiograph, sputum smear microscopy, histopathology, and molecular tests) and treatment outcome using the Statistical Package for the Social Sciences (SPSS) version 25.

#### Time series

The times series is characterized as a sequential collection of observations indexed over time [16]. Hence, the TB cases reported by the prison system and grouped as mentioned earlier were organized according to the month they were reported to obtain monthly rates.

The Prais-Winsten self-regression method [17] was performed using Software for Statistics and Data Science (STATA) version 14 to classify the event’s time trend into ascending, descending, or stationary in the period. The monthly percent change (MPC) was calculated whenever a time trend was classified as ascending or descending, along with its respective 95% confidence interval (95%CI) [16]. Note that a time trend refers to the ascending, descending, or stationary tendency of a time series in a given period [16, 18].

Then, the robust Seasonal-Trend by Loess (STL by Loess) [18] method was used. This decomposition method is based on a locally weighted regression (Loess), so it is used to estimate non-linear relationships, separating the components of a time series into trend, seasonality, or noise [18, 19]. Rstudio was used with the forecast package.

Opposed to the Prais-Winsten method, in which the time trend is globally assessed and a constant is generated to classify the entire period, the STL by Loess method assesses the time trend over the period, verifying its variations over time and whether the trend was always ascending/descending or stationary or there were variations with peaks and/or decreases.

### Spatial analysis

The Getis-Ord Gi* statistic, a technique consisting of a local indicator of spatial association, was used to identify the social determinants and cluster formation in each prison in the state based on a neighbourhood matrix. A z-score was generated for the statistically significant cities; the larger the z-score was, the more intense the clustering of high values (hot spot). The same logic was used for a negative z-score (i.e., the smaller the z-score was, the more intense the clustering of low values (cold spot)).

In addition to the z-score, the *p*-value and significance level (Gi-Bin) determine whether hot spots and cold spots are statistically significant. For example, values may range between ± 3 and reflect statistical significance with a 99% confidence level between ± 2 with a 95% confidence level and ± 1 with a 90% confidence level; zero corresponds to statistically insignificant areas.

Spatial scan statistics were used to identify the TB risk spatial areas for the incarcerated population in Paraná, Brazil. First, spatial clusters were identified by finding circles with a variable radius around each centre of the cities with prison facilities. Then, the number of observed and expected cases within each circle was calculated, and this procedure was performed until all centres were tested. A cluster was identified when the value observed in the area within the circle was greater or smaller than the expected [20].

In this stage, the following characteristics were considered: Poisson’s discrete model, only high-risk clusters, no overlapping geographic clusters, circular clusters, and 999 Monte Carlo simulation, while the size of the population exposed was determined by the Gini coefficient in which the number of expected cases in each state is proportional to the size of the population at risk [20, 21]. The relative risk (RR) and 95%CI of each cluster were also calculated; statistical significance was established at *p* < 0.05.

### Ethical aspects

The study was approved by the Research Ethics Committee at the University of São Paulo at Ribeirão Preto College of Nursing under Certificate of Presentation for Ethical Consideration number 24963319.1.0000.5393 and report number 3.836.401 issued on 13 February 2020 in accordance with the Guidelines and Regulatory Standards for Research with Human Subjects, Resolution number 466/2012 of the National Health Council of Brazilian Ministry of Health.

## Results

A total of 1,099 TB cases were reported among the incarcerated population in Paraná. The use of tobacco was the most frequently reported (*n* = 460; 41.8%; 95%CI: 0.38–0.44), followed by illegal drugs (*n* = 451; 41.0%; 95%CI: 0.38–0.43) and alcohol (*n* = 179; 16.3%; 95%CI: 014–018). Regarding sociodemographic characteristics, most were male individuals aged between 18 and 29 years old with a low educational level (< 8 years of schooling). When analyzing the variables by type of drug, it was possible to verify that the sociodemographic characterization of the studied population follows the same profile, the male gender, caucasian, age group between 18 and 29 years and schooling from 5 to 8th grade were the more prevalent among the population that consumes alcohol, tobacco and other drugs (Table 1).

**Table 1:** Sociodemographic characteristics of incarcerated population diagnosed with TB according to the type of drug used and total incarcerated population, Paraná, Brazil. (*N* = 1,099)

Variables	Incarcerated population using alcohol	Incarcerated population using tobacco	Incarcerated population using illegal drugs	Total incarcerated population
	**n (%)**	**n (%)**	**n (%)**	**n (%)**
**Gender**				
Male	173(96.6)	447(97.2)	440(97.6)	1064(96.8)
Female	6(3.4)	13(2.8)	11(2.4)	35(3.2)
**Age group (years)**				
18 to 29	78(43.6)	253(55.0)	269(59.6)	614(55.9)
30 to 39	64(35.8)	142(30.9)	138(30.6)	326(29.7)
40	37(20.7)	64(13.9)	40(9.5)	156(14.2)
**Race**				
Caucasian	112(62.6)	280(60.9)	290(64.3)	722(65.7)
Mixed race	48(26.8)	136(29.6)	122(27.1)	275(25.0)
African descent	19(10.6)	37(8.0)	34(7.5)	83(7.6)
Asian descent	-	3(0.7)	1(0.2)	3(0.3)
Indigenous	-	-	-	2(0.2)
Ignored	-	4(0.8)	2(0.4)	14(1.3)
**Education**				
Illiterate	9(5.0)	14(3.0)	8(1.8)	22(2.0)
1st to 4th grade	35(19.6)	96(20.9)	78(17.3)	198(18.0)
5th to 8th grade	82(45.8)	222(48.9)	240(53.2)	564(51.3)
> 8 years	32(17.9)	77(16.7)	62(13.7)	297(27.0)

Sources: study’s data

TB/HIV co-infection was more prevalent among smokers and those using illegal drugs, whereas TB/diabetes mellitus and mental disorders were more frequent among those consuming alcohol. The TB clinical profile included new cases, pulmonary TB, no radiograph, positive sputum smear microscopy, histopathology, and molecular test revealing sensitivity to rifampicin among the groups using tobacco and illegal drugs. Regarding TB treatment outcome, cure and DR-TB were predominant among smokers and individuals using illegal drugs, while alcohol consumers tended to abandon the treatment more frequently (Table 2).

**Table 2:** Clinical characteristics of incarcerated population diagnosed with TB using alcohol, tobacco, or illegal drugs. Paraná, Brazil (*N* = 1,099)

Variables	Incarcerated population using alcohol	Incarcerated population using tobacco	Incarcerated population using illegal drugs	Total incarcerated population
	**n (%)**	**n (%)**	**n (%)**	**n (%)**
**AIDS**				
Yes	21(11.7)	39(8.5)	39(8.6)	82(7.5)
No	155(86.6)	409(88.9)	394(87.4)	971(88.4)
**Diabetes Mellitus**				
Yes	7(3.9)	13(2.8)	8(1.8)	21(1.9)
No	170(95.0)	430(93.5)	422(93.6)	1025(93.3)
**Mental disorder**				
Yes	4(2.2)	9(2.0)	8(1.8)	17(1.5)
No	172(96.1)	435(94.6)	424(94.0)	1029(93.6)
**Admission**				
New case	136(76.0)	359(78.0)	344(76.3)	880(80.1)
Relapse	11(6.1)	29(6.3)	30(6.7)	70(6.4)
Retreatment after treatment abandonment	20(11.2)	38(8.3)	39(8.6)	66(6.0)
Transference	12(6.7)	34(7.4)	38(8.4)	79(7.2)
**Type**				
Pulmonary	156(87.2)	410(89.1)	402(89.1)	968(88.1)
Extrapulmonary	21(11.7)	40(8.7)	39(8.6)	105(9.6)
Pulmonary + extrapulmonary	2(1.1)	10(2.2)	10(2.2)	26(2.4)
**Radiograph**				
Suspected TB	148(82.7)	374(81.3)	360(79.8)	877(79.8)
Normal	3(1.7)	10(2.2)	9(2.0)	32(2.9)
Other pathology	1(0.6)	-	-	3(0.3)
Not taken	26(14.5)	75(16.3)	80(17.7)	182(16.6)
**Sputum smear microscopy**				
Positive	114(63.7)	288(62.6)	302(67.0)	703(64.0)
Negative	35(19.6)	80(17.4)	65(14.4)	179(16.3)
Not taken	25(14.0)	86(18.7)	78(17.3)	202(18.4)
**Histopathology**				
Baar Positive	15(8.4)	16(3.5)	22(4.9)	69(6.3)
Suggestive of TB	5(2.8)	21(4.6)	19(4.2)	44(4.0)
Not suggestive of TB	3(1.7)	4(0.9)	4(0.9)	8(o.7)
Not performed	153(85.5)	405(88.0)	396(87.8)	951(86.5)
**Molecular test**				
Rifampicin sensitivity detected	65(36.3)	227(49.3)	223(49.4)	432(39.3)
Rifampicin resistance detected	3(1.7)	11(2.4)	9(2.0)	21(1.9)
Not detectable	9(5.0)	35(7.6)	29(6.4)	57(5.2)
Not performed	97(54.2)	179(38.9)	182(40.4)	543(49.4)
**Outcome**				
Cure	116(64.8)	312(67.8)	301(66.7)	743(67.6)
Abandoned	25(14.0)	42(9.1)	40(8.9)	89(8.1)
Death due to TB	2(1.1)	4(0.9)	3(0.7)	18(1.6)
Death due to another cause	5(2.8)	15(3.3)	9(2.0)	31(2.8)
Transferred	18(10.1)	43(9.3)	51(11.3)	119(10.8)
DR-TB	12(6.7)	41(8.9)	43(9.5)	75(6.8)

Source: Study’s data

The time trend of TB cases in the total incarcerated population was classified as descending, with a decrease of 49.8%/mo. (95%CI: -35.43 to -60.19). When analysing the psychotic substances, an ascending time trend was found for alcohol consumers (+ 19.4%/mo. (95%CI: 12.20–23.03)); smokers (+ 20.2% (95%CI: 12.20–28.82); and drug users (+ 62.2%/mo. (95%CI: 44.54–81.97)), as shown in Table 3.

**Table 3:** Time trend of TB incidence among incarcerated population according to the consumption of psychoactive substances. Paraná, Brazil. (2014–2018). (*N* = 1,099)

**Variable**	**Coefficient**	**(95%CI)**^a^	**Time trend**	**MPC**^b^ **(95%CI)**
Total incarcerated population	-0.30	(-0.19—-0.40)	Descending	-49.88 (-35.43—60.19)
Incarcerated population consuming alcohol	0.07	(0.05 – 0.09)	Ascending	19.40 (12.20 – 23.03)
Incarcerated population using tobacco	0.08	(0.05 – 0.11)	Ascending	20.23 (12.20 – 28.82)
Incarcerated population using illegal drugs	0.21	(0.16 – 0.26)	Ascending	62.18 (44.54 – 81.97)

Source: Study’s data

^a^*95%CI* 95% Confidence Interval

^b^*MPC* Monthly percentage change

**Fig. 2:**
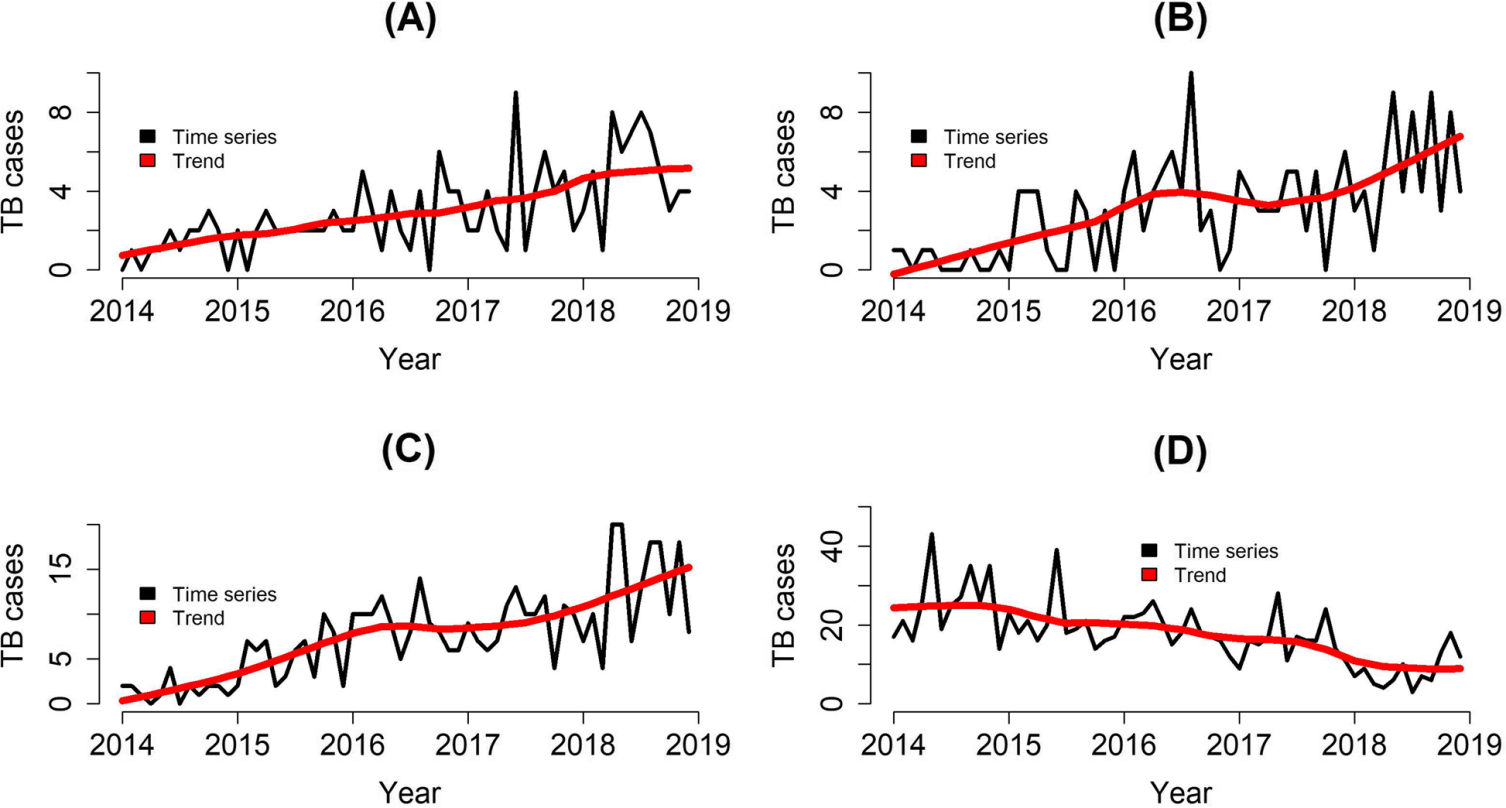
TB time series concerning incarcerated population in prisons located in Paraná, Brazil (2014–2019) (*N* = 1,099)

The time series decomposition technique (Fig. 2A, B, and C) showed an increase in the TB time trend among the incarcerated population using alcohol, tobacco, and illegal drugs, whereas Fig. 2D shows a decrease in the time trend of the total population between 2014 and 2018. These findings corroborate the data presented in Table 3 concerning the results from the Prais-Winsten analysis.

**Fig. 3:**
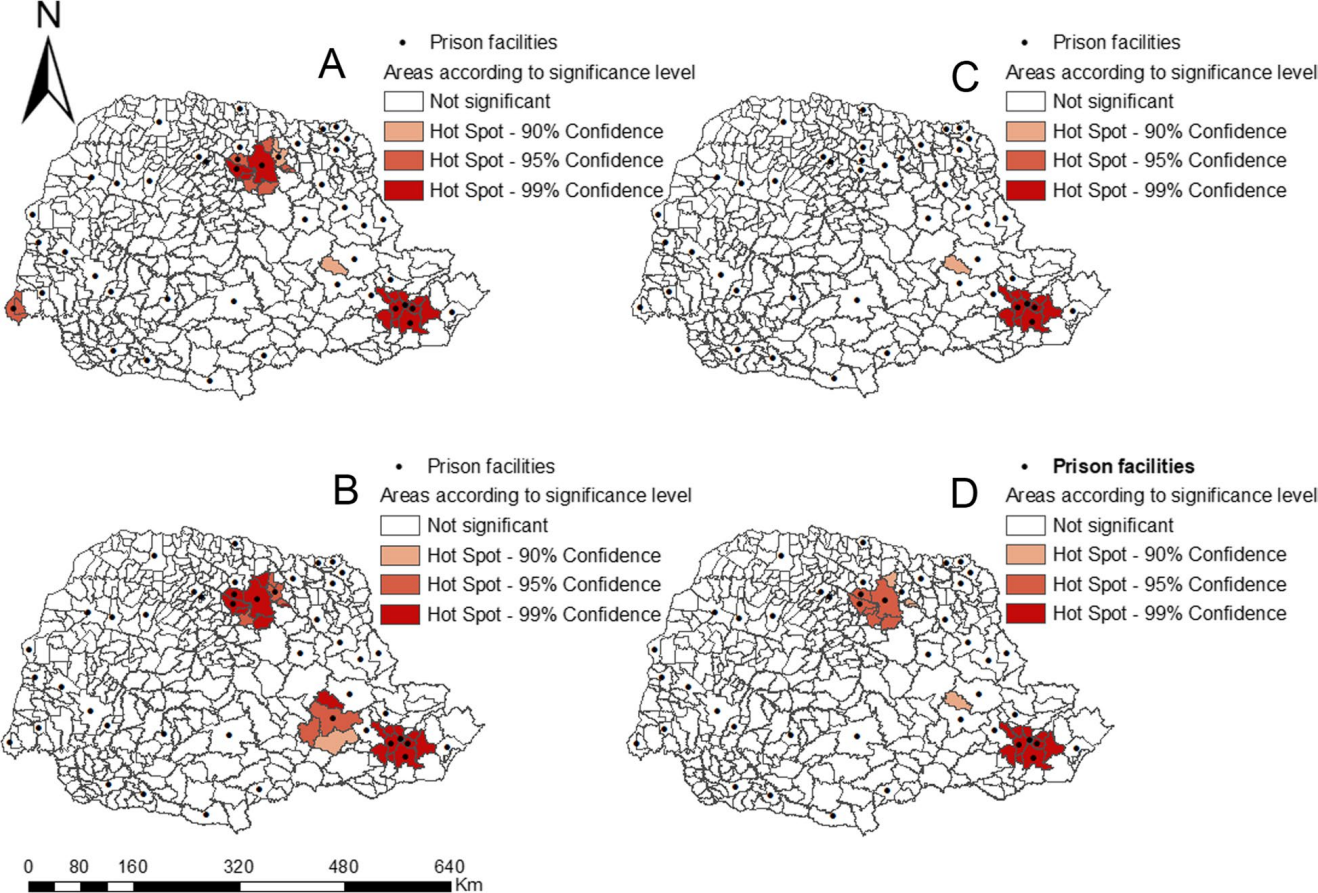
TB high and low clusters for the prison facilities. Paraná, Brazil, 2014–2018

Figure 3A concerns the total incarcerated population. The Getis-Ord Gi* technique identified three hot spots: east (metropolitan region of Curitiba), north and west macro-regions. Figure 3B reveals three hot spots: two in the east macro-region (metropolitan region of Curitiba and in Ponta Grossa) and one in the north macro-region; the pseudo-significance test presented a z-score of 4.27 confirming the non-randomness of the clusters (*p* < 0.00). Figure 3C shows a hot spot in the east macro-region (z-score = 2.41 and *p* < 0.01), and Fig. 3D presents two hot spots in the east and north macro-regions (z-score = 2.13 and *p* < 0.03).

**Fig. 4:**
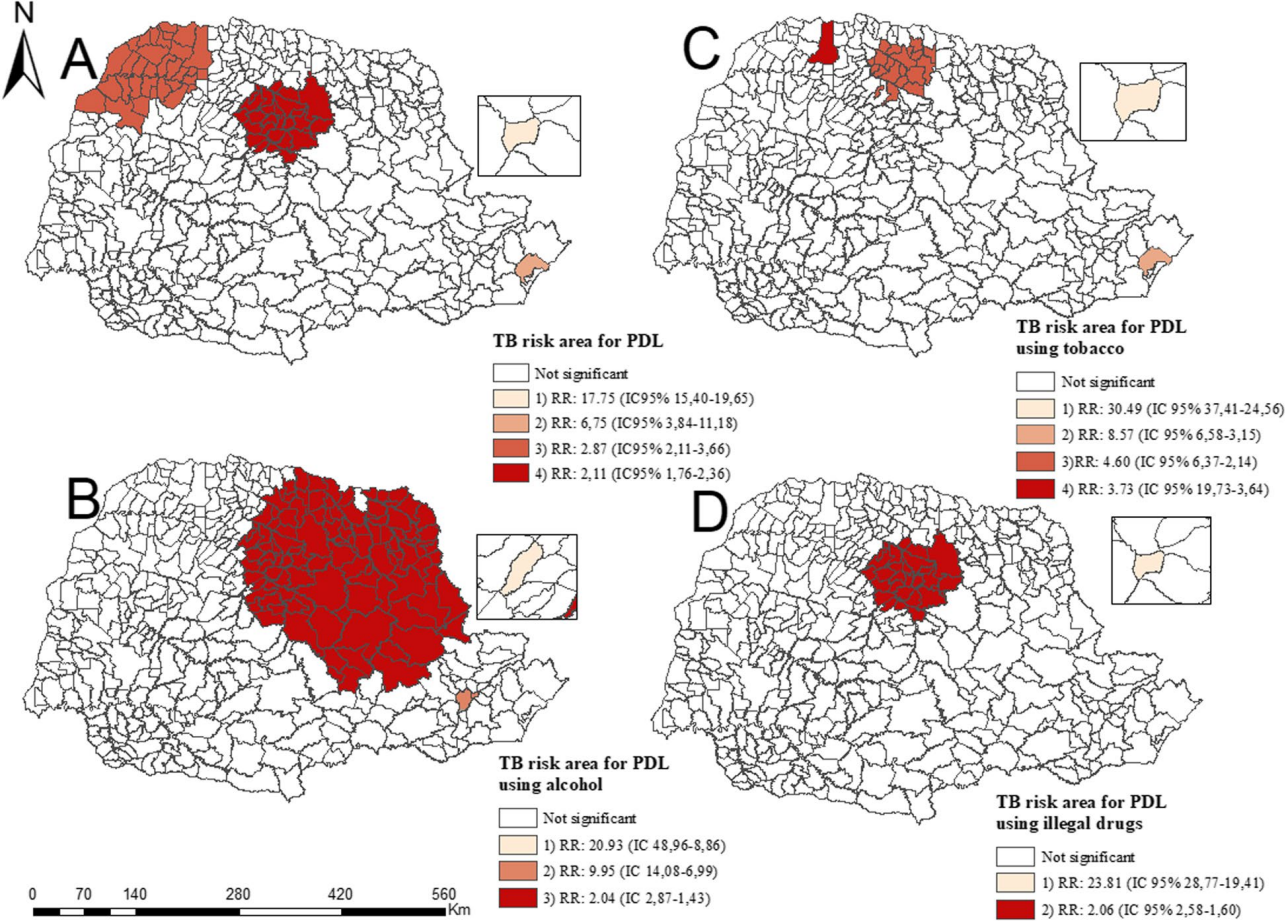
Areas of spatial risk for tuberculosis among incarcerated population users of alcohol, tobacco and other drugs. Paraná, Brazil, 2014–2018

Figure 4A shows a relationship between TB and the total incarcerated population, indicating four spatial risk clusters, namely: 1) one in the city of Pinhais located in the east macro-region (metropolitan region of Curitiba) (RR: 17.75 (95%CI 15.40–19.65)) with 832 individuals in the incarcerated population and 296 cases observed; 2) in the city of Paranaguá located in the east macro-region (Paraná coast region) (RR: 6.75 (95%CI 3.84–11.18)) with 56 individuals in the incarcerated population and five cases observed; 3) in the north macro-region (RR: 2.87 (95%CI 2.11–3.66)) with 616 individuals in the incarcerated population and 50 cases observed; and 4) one in the northwest macro-region (RR: 2.11 (95%CI 1.76–2.36)) with 3,911 individuals in the incarcerated population and 212 cases observed.

Figure 4B presents three risk clusters among the incarcerated population diagnosed with TB using alcohol, namely: 1) one in the city of Ourizona located in the northwest macro-region presenting (RR: 20.93 (95%CI 8.86–48.96)) with 56 individuals in the incarcerated population and five cases reported; 2) one located in the east macro-regional in the metropolitan region of Curitiba (RR: 9.95 (95%CI 6.99–14.08)) with 1,670 individuals in the incarcerated population and 49 cases reported; and 3) one cluster located in the north macro-region (RR: 2.05 (95%CI 1.43–2.87)) with 7,219 individuals in the incarcerated population and 52 cases reported.

Figure 4C presents four spatial risk clusters concerning the incarcerated population using tobacco, namely: 1) one in the city of Pinhais in the east macro-region (metropolitan region of Curitiba) (RR: 30.49 (95%CI 24.56–37.41)) with 832 individuals in the incarcerated population and 142 cases reported; 2) one in Paranaguá in the east macro-region (Paraná’s coast region) (RR: 8.57 (95%CI 3.15–6.58)) with 56 individuals in the incarcerated population and five cases observed; 3) one in the north macro-region (RR:4.60 (95%CI 2.14–6.37)) with 667 individuals in the incarcerated population and 30 cases observed; and 4) one in Paranavaí in the northwest macro-region (RR:3.73 (95%CI 3.64–19.73)) with 264 individuals in the incarcerated population and nine cases observed.

Figure 4D presents two risk clusters of the incarcerated population using drugs, namely: 1) in the city of Pinhais in the east macro-region (metropolitan region of Curitiba) (RR:23.81 (95%CI 19.41–28.77)) with 832 individuals in the incarcerated population and 149 cases reported and 2) one located in the north macro-region (RR:2.06 (95%CI 1.60–2.58)) with 3,911 individuals in the incarcerated population and 87 cases reported.

## Discussion

The use of tobacco and illegal drugs was prevalent among the incarcerated population diagnosed with TB, and an ascending trend was verified for alcohol, tobacco, and illegal drugs in prison facilities. This study contributes to the scientific technical improvement in the area, since most studies address TB among the incarcerated population; thus, there is a knowledge gap when associating TB among users of alcohol, tobacco and other drugs.

The highest RR was found in the prisons located in the east macro-region (metropolitan region of Curitiba), showing that prisons contribute to the TB burden and are an environment that favours the disease, considering a large number of alcohol consumers and drug users [6, 8, 11, 22, 23].

Note that the medical centre providing care to prisoners is located in the east macro-region, which explains the high number of TB cases in this area. Data suggest that the availability of medical services indicates the incarcerated population has access to healthcare services, and the high rates of outpatient care consultations and hospitalizations suggest the quality of the health conditions of these individuals [24].

It is worth noting that, in 2003, the Ministry of Health, together with the Ministry of Justice, launched the National Health Plan for the Prison System and, in 2014, established the National Policy for Integral Health Care for Persons Deprived of Liberty in the Prison System to promote health and prevent diseases in the prison system and ensure the incarcerated population has access to integral and quality healthcare provided by the Brazilian Unified Health System [25].

There are many factors that lead individuals to incarceration throughout their lives, but it can be highlighted that the conditions and social exclusion are an extremely relevant characteristic for triggering acts that lead to deprivation of liberty [26].

However, it is worth mentioning that incarcerated population are considered to be a population that is at high risk for illness and the development of different comorbidities, including TB, due to their living conditions and exposure to unfavorable health behaviors such as alcohol use, tobacco and other drugs [10xx].

Another characteristic that influences the development of TB among incarcerated population who use alcohol, tobacco and other drugs is the very lifestyle that significantly contributes to TB. A study points out that socioeconomic, social and political conditions, as well as assistance to health services and the distribution of social determinants are factors that directly influence the increase in the incidence of TB within a territory [27].

Thus, it is noted the fundamental importance that governments have in the face of two major social and public health problems, which is to invest in protective policies that distance young people from crime and offer educational support and living conditions as well as effective health services. for the control and elimination of TB in the world.

In view of this scenario, a high number of TB cases have been observed among the incarcerated population, mainly pulmonary TB, which is of concern considering that the bacillus is airborne and the conditions of the prisons, such as overcrowding and poor ventilation, favour the dissemination of TB [28]. Therefore, efficient public policies are intended to provide healthcare to the incarcerated population.

The incarcerated population includes socially vulnerable people affected by different diseases, [25] among which dependency on alcohol, tobacco, and illegal drugs stand out. Even though alcohol and tobacco consumption have decreased in the population in general, there was a significant increase in the number of smokers and drug users among the incarcerated population [22]. Studies report that approximately 80% of the incarcerated population in the United States have a history of illegal drug use [23]. One study was conducted in Norway to investigate drinking habits before imprisonment and reported that 55% of the prisoners had alcohol problems of some severity; 18% of them were possibly alcohol dependent [29]. Not much data are available in Brazil, and research in the field is still incipient considering the difficulty in discussing this phenomenon in public security institutions [30].

Hence, the consumption of legal or illegal psychoactive substances within prisons contributes to an increase in the number of diseases, and as this study shows, contributes to the development and maintenance of TB [31, 32]. Furthermore, these substances not only contribute to the development of TB but also lead to unfavourable treatment outcomes, considering that psychoactive substances are associated with higher rates of mental disorders, suicide, mortality, relapse after release [33], and violence within prisons.

The consumption of PS favours the development of diseases among the incarcerated population and is associated with higher rates of physical violence and suicide attempts within prisons; suicide attempts in this population are estimated to be three to eight times higher than in the general population. Risk factors include mental disorders, substance use disorders, suicidal ideation, suicide attempts, self-injury behaviours, accommodation in single-occupancy cells, and conviction due to violent crimes [34].

The social and spatial factors of prisons directly contribute to the maintenance of TB and other diseases, considering many prisons are overcrowded, present high turnover of the incarcerated population, are poorly ventilated, and have restricted access to health services [35].

The incarcerated population live in an unhealthy environment with poor hygiene conditions, which often fail to ensure basic human needs to protect one’s physical and mental health, directly contributing to disseminating transmissible diseases, violence-related injuries, and mental disorders [36]. These are characteristics observed in the east, north, and northwest macro-regions, which host risk areas for total TB and TB associated with alcohol, tobacco, and illegal drugs. Note that the west macro-region was not risk area for TB associated with psychoactive substances.

The identification of risk prison facilities for the development of TB associated with the consumption of psychoactive substances can support the implementation of preventive measures and quality healthcare, especially in those facilities with a large number of prisoners, that is, facilities exposed to a higher risk. However, there is usually a delay in the diagnosis of TB, with a high prevalence of resistant bacteria; inadequate treatment and treatment abandonment; low educational level; malnutrition; mental disorders; previous diseases; TB/HIV co-infection; and alcohol, tobacco, and/or illegal drug consumption/dependency [6, 7, 37].

Therefore, prisons are a reservoir of various diseases, especially infectious-contagious diseases, such as TB. The community is also exposed to TB when coming into contact with prison officers, released prisoners, or families visiting prisoners. Hence, the prison environment promotes the incidence and maintenance of TB, and health actions are needed to break the transmission cycle and decrease the number of new cases and deaths [35].

Time trend analysis showed TB increased among the incarcerated population consuming alcohol, tobacco, or illegal drugs, which is similar in the general population. The fact that the consumption of these substances has increased worldwide is of concern, considering it is associated with worsened TB treatment outcomes [24, 28].

Therefore, a screening protocol should be implemented in the prison system to identify the consumption of psychoactive substances and TB and invest and give priority to early diagnosis and interventions, providing appropriate treatment to avoid interruptions and relapses [35].

Achieving the goals established by the End TB Strategy and eradicating TB by 2050 will only be possible by investing in preventive measures and appropriate TB treatment. Therefore, one of the most difficult challenges is to control the progress of the disease among subpopulations presenting high incidence rates, such as the incarcerated population [38]. In this sense, the incarcerated population is a social stratum at a higher risk for TB, [8] and strategies are needed to decrease the transmission of the disease and achieve the global goals.

In this sense, the WHO recommends measures be implemented in prisons to prevent new cases, including screening protocols applied in the admission and discharge of inmates, in addition to periodic assessments of the incarcerated population or isoniazid preventive therapy [2]. Routinely screening prisoners, isolating confirmed cases, decreasing the number of inmates in a single cell, and avoiding agglomerations are efficient ways to decrease transmission among prisoners, prison officers, families, and the community [8].

Programs intended to obtain early diagnoses, the report of new cases, and the implementation of proper treatment are vital. However, the access of the incarcerated population to health services is restricted, resulting in unfavourable outcomes and high rates of TB.

## Conclusions

This study advances the knowledge in the field, as it shows the consumption of alcohol, tobacco, and illegal drugs among the incarcerated population diagnosed with TB. Clustering was found in some areas in the state with an ascending trend, highlighting the need to implement policies to control the disease in vulnerable areas and areas hosting prisons. Even though there are studies addressing TB in the prison system, this is one of the first studies investigating spatial differences and determinants using geostatistics.

These techniques are seldom adopted to address these populations, showing the originality and relevance of this investigation. Decreasing disparities and inequalities involve understanding the burden of the disease and its determinants in the prison system, so innovative approaches and studies are also needed.

## Data Availability

All the data supporting the study findings are within the manuscript. Additional detailed information and raw data will be shared upon request addressed to the corresponding author.

## Abbreviations

CI: Confidence Interval
DR-TB: Drug-Resistant Tuberculosis
HIV: Human Immunodeficiency Virus
MPC: Monthly Percent Change
RR: Relative Risk
SINAN: Notifiable Diseases Information System
SPSS: Statistical Package for the Social Sciences
STATA: Software for Statistics and Data Science
STL: Seasonal-Trend by Loess
TB: Tuberculosis
WHO: World Health Organization

## References

[1] Seki M, Choi H, Kim K, Whang J, Sung J, Mitarai S (2021). Tuberculosis: a persistent unpleasant neighbour of humans. J Infect Public Health.

[2] World Health Organization. Global Tuberculosis Report 2020. Geneva, Switzerland, 2020 Available from: https://apps.who.int/iris/bitstream/handle/10665/336069/9789240013131-eng.pdf

[3] Visca D, Ong CWM, Tiberi S, Centis R, D'Ambrosio L, Chen B (2021). Tuberculosis and COVID-19 interaction: a review of biological, clinical and public health effects. Pulmonology.

[4] Brugueras S, Molina VI, Casas X, González YD, Forcada N, Romero D (2020). Tuberculosis recurrences and predictive factors in a vulnerable population in Catalonia. PLoS One.

[5] Ministry of Health. Health Surveillance Secretariat. Epidemiological Bulletin, Brasília. Special Issue. March 2021. 1–43. Available from: https://www.gov.br/saude/pt-br/media/pdf/2021/marco/24/boletim-tuberculose-2021_24.03

[6] Chekesa B, Gumi B, Chanyalew M, Zewude A, Ameni G (2020). Prevalence of latent tuberculosis infection and associated risk factors in prison in East Wollega Zone of western Ethiopia. PLoS One.

[7] Fuge TG, Ayanto SY (2016). Prevalence of smear positive pulmonary tuberculosis and associated risk factors among prisoners in Hadiya Zone prison. Southern Ethiopia BMC Res Notes.

[8] Cords O, Martinez L, Warren JL, O’Marr JM, Walter KS, Cohen T (2021). Incidence and prevalence of tuberculosis in incarcerated populations: a systematic review and meta-analysis. Lancet Public Health.

[9] Lima MCRAA, Martinez-Marcos MM, Ballestero JGA, Weiller TH, Oliveira CBB, Palha PF (2021). Control de la tuberculosis en un sistema penitenciario brasileño: un estudio con métodos mixtos. Anna Nery Sch J Nurs.

[10] Gatechompol S, Harnpariphan W, Supanan R, Suwanpimolkul G, Sophonphan J, Ubolyam S, Kerr SJ, Avihingsanon A, Kawkitinarong K (2021). Prevalence of latent tuberculosis infection and feasibility of TB preventive therapy among Thai prisoners: a cross-sectional study. BMC Public Health.

[11] Nogueira PA, Abrahão RMCM, Galesi VMN, López RVM. Tuberculosis and latent infection in employees of different prison unit types. Rev Saude Publica. 2018;52:13. 10.11606/S1518-8787.2018052007127 10.11606/S1518-8787.2018052007127 PMC5802647 29412377

[12] Rothman KJ, Greenland S, Lash TL (2008). Modern Epidemiology.

[13] National Penitentiary Department. National Prison Information Survey. Period from July 2019 to December 2019. Available from: https://app.powerbi.com/view?r=eyJrIjoiMmU4ODAwNTAtY2IyMS00OWJiLWE3ZTgtZGNjY2ZhNTYzZDliIiwidCI6ImViMDkwNDIwLTQ0NGMtNDNmNy05MWYyLTRiOGRhNmJmZThlMSJ9

[14] Palmeira JLM, Vasconcelos KIR, Lima KA, Santana JS, Santos L, Vasconcelos JMB. Tuberculosis treatment from the perspective of incarcerated individuals in a maximum security unit R. Pesq Cuid Fundam Online. 2021;13:907–11. 10.9789/2175-5361.rpcfo.v13.9614

[15] World Drug Report. Booklet 2 - Global overview of drug demand and drug supply. 2021. Available from: https://www.unodc.org/unodc/en/data-and-analysis/wdr2021.html

[16] Antunes JLF, Cardoso MRA (2015). Using time series analysis in epidemiological studies. Epidemiol Serv Saude.

[17] Prais SJ, Winsten CB. Trend Estimates and Serial Correlation. Cowles Commission Discussion Paper: Statistics No. 383. 1954;1-26.

[18] Cleveland RB, Cleveland WS, McRae JE, Terpenning I (1990). STL: a seasonal-trend decomposition procedure based on loess. J Off Stat.

[19] Brockwell PJ, Davis RA (2002). Introduction to Time Series and Forecasting.

[20] Kulldorff M (2015). SaTScan User Guide V9.4. SaTScan TM User Guid version 94.

[21] Han J, Zhu L, Kulldorff M, Hostovich S, Stinchcomb DG, Tatalovich Z (2016). Using Gini coefficient to determining optimal cluster reporting sizes for spatial scan statistics. Int J Health Geogr.

[22] Woodall J, Tattersfield A (2018). Perspectives on implementing smoke-free prison policies in England and Wales. Health Promot Int.

[23] Rowell-Cunsolo TL, Sampong SA, Befus M, Mukherjee DV, Larson EL (2016). Predictors of Illicit drug use among prisoners. Subst Use Misuse.

[24] Kouyoumdjian FG, Cheng SY, Fung K, Orkin AM, McIsaac KE, Kendall C (2018). The health care utilization of people in prison and after prison release: a population-based cohort study in Ontario, Canada. PLoS One.

[25] Ministry of Health. Health Care Secretariat. Department of Strategic Programmatic Actions. Health Coordination in the Prison System. National Policy for Comprehensive Health Care for Persons Deprived of Liberty in the Prison System (PNAISP). Available from: http://www.as.saude.ms.gov.br/wp-content/uploads/2016/06/Cartilha-PNAISP.pdf

[26] Honorato B, Caltabiano N, Clough AR (2016). From trauma to incarceration: exploring the trajectory in a qualitative study in male prison inmates from north Queensland. Australia Health Justice.

[27] Duarte R, Lönnroth K, Carvalho C, Lima F, Carvalho ACC, Muñoz-Torrico M, Centis R (2018). Tuberculosis, social determinants and co-morbidities (including HIV). Pulmonology.

[28] Alves KKAF, Borralho LM, Araújo AJ, Bernardino IM, Figueiredo TMRM (2020). Factors associated with recovery and the abandonment of tuberculosis treatment in the incarcerated population. Rev Bras Epidemiol.

[29] Pape H, Rossow I, Bukten A (2021). Alcohol problems among prisoners: subgroup variations, concurrent drug problems, and treatment needs. Eur Addict Res.

[30] Dalmaso TF, Meyer DEE (2017). Drug circulation and consumption in a female penitentiary: perceptions of a prison health team. Saúde debate.

[31] Young JT, Puljević C, Love AD, Janca EK, Segan CJ, Baird D (2019). Staying Quit After Release (SQuARe) trial protocol: a randomised controlled trial of a multicomponent intervention to maintain smoking abstinence after release from smoke-free prisons in Victoria, Australia. BMJ Open.

[32] Winkelman TNA, Vickery KD, Busch AM (2019). Tobacco use among non-elderly adults with and without criminal justice involvement in the past year: United States, 2008–2016. Addict Sci Clin Pract.

[33] Baranyi G, Scholl C, Fazel S, Patel V, Priebe S, Mundt AP (2019). Severe mental illness and substance use disorders in prisoners in low-income and middle-income countries: a systematic review and meta-analysis of prevalence studies. Lancet Glob Health.

[34] Larney S, Topp L, Indig D, O'Driscoll C, Greenberg D (2012). A cross-sectional survey of prevalence and correlates of suicidal ideation and suicide attempts among prisoners in New South Wales. Australia BMC Public Health.

[35] Sequera VG, Aguirre S, Estigarribia G, Cellamare M, Croda J, Andrews JR (2020). Increased incarceration rates drive growing tuberculosis burden in prisons and jeopardize overall tuberculosis control in Paraguay. Sci Rep.

[36] Job Neto F, Miranda RB, Coelho RA, Gonçalves CP, Zandonade E, Miranda AE (2019). Health morbidity in Brazilian prisons: a time trends study from national databases. BMJ Open.

[37] Allgayer MF, Ely KZ, Freitas GH, Valim ARM, Gonzales RIC, Krug SBF (2019). Tuberculosis: health care and surveillance in prisons. Rev Bras Enferm.

[38] Mabud TS, Lourdes Delgado Alves M, Ko AI, Basu S, Walter KS, Cohen T (2019). Evaluating strategies for control of tuberculosis in prisons and prevention of spillover into communities: an observational and modeling study from Brazil. PLoS Med.

